# Case Report: Dramatic metabolic improvement with tirzepatide in a patient with acquired partial lipodystrophy following hematopoietic stem cell transplantation

**DOI:** 10.3389/fendo.2026.1868458

**Published:** 2026-06-19

**Authors:** Yuri Tanaka, Hiroaki Ueno, Hirotake Sawada, Ayaka Konagata, Taisuke Uchida, Yudai Uehira, Fumiko Kogo, Akari Sekishima, Hiroshi Moritake, Hideki Yamaguchi, Kazuya Shimoda

**Affiliations:** 1 Division of Hematology, Diabetes, and Endocrinology, Department of Internal Medicine, Faculty of Medicine, University of Miyazaki, Kiyotake, Miyazaki, Japan; 2 Division of Pediatrics, Faculty of Medicine, University of Miyazaki, Miyazaki, Japan; 3 Department of Nursing, Faculty of Medicine University of Miyazaki, Miyazaki, Japan

**Keywords:** adiponectin, dual incretin receptor agonism, hematopoietic stem cell transplantation, insulin resistance, lipodystrophy, tirzepatide

## Abstract

**Background:**

Acquired lipodystrophy is a rare disorder characterized by adipose tissue loss or dysfunction and is frequently associated with severe insulin resistance and metabolic complications. Metabolic complications of lipodystrophy have occasionally been reported after hematopoietic stem cell transplantation (HSCT), but their clinical features and optimal treatment strategies remain poorly defined. Tirzepatide, a dual agonist of the glucose-dependent insulinotropic polypeptide (GIP) and glucagon-like peptide-1 (GLP-1) receptors, has recently emerged as a novel therapy for type 2 diabetes.

**Case presentation:**

We report a 37-year-old woman who underwent allogeneic HSCT at 12 years of age for relapsed acute lymphoblastic leukemia after a conditioning regimen including total body irradiation (TBI), high-dose cytarabine, and melphalan. She subsequently developed diabetes mellitus and fatty liver disease at 17 years of age. Although no apparent fat loss was initially recognized, lipodystrophy was clinically suspected based on severe insulin resistance and metabolic abnormalities disproportionate to her body habitus. Computed tomography at 37 years of age revealed region-specific fat loss extending from the lower back to the gluteal region. Glycemic control remained inadequate despite high-dose insulin therapy and sequential treatment with several GLP-1 receptor agonists. After initiation of tirzepatide, glycemic control improved dramatically, allowing complete discontinuation of insulin therapy. Body weight decreased modestly and hepatic steatosis improved. The high-molecular-weight (HMW)/total adiponectin ratio after treatment was elevated (64.0%), suggesting possible improvement in adipocyte secretory function.

**Conclusion:**

This case highlights acquired partial lipodystrophy developing after HSCT, supported by region-specific fat loss and characteristic metabolic abnormalities, and demonstrates a marked therapeutic response to tirzepatide. Dual incretin receptor agonism may represent a promising therapeutic strategy for severe insulin resistance associated with adipose tissue dysfunction, potentially through both weight-dependent and weight-independent mechanisms.

## Introduction

Lipodystrophy comprises a heterogeneous group of disorders characterized by loss or dysfunction of adipose tissue independent of energy intake and is frequently associated with severe insulin resistance, diabetes mellitus, hypertriglyceridemia, and fatty liver disease (1–3). It may be congenital or acquired and can manifest in generalized, partial, or localized forms. Acquired partial lipodystrophy is a rare disorder characterized by region-specific loss of subcutaneous adipose tissue and is often accompanied by marked metabolic abnormalities disproportionate to body mass (1, 3). Some acquired cases are known to occur secondary to immunological alterations, including autoimmune diseases and hematopoietic stem cell transplantation (HSCT) (3).

Various autoimmune complications may develop after HSCT as a result of immune reconstitution or graft-versus-host disease (GVHD) (4). However, reports of lipodystrophy following transplantation remain limited (5–10). Among the reported cases, no consistent patterns have been observed with respect to the timing of onset, the distribution of fat loss, or associated metabolic abnormalities, and the underlying pathophysiological mechanisms remain unclear.

Diabetes mellitus associated with partial lipodystrophy is characterized by severe insulin resistance and is often difficult to control despite high-dose insulin therapy. In recent years, several reports have described the efficacy of glucagon-like peptide-1 (GLP-1) receptor agonists in this context (11, 12). Tirzepatide, a dual agonist targeting both the GLP-1 receptor and the glucose-dependent insulinotropic polypeptide (GIP) receptor, has been suggested to exert unique effects on insulin sensitivity and adipose tissue metabolism (13, 14). Very recently, its efficacy has also been reported in patients with diabetes associated with congenital generalized lipodystrophy (15) as well as familial partial lipodystrophy (16).

We report a patient with acquired partial lipodystrophy that developed after HSCT and subsequent GVHD, who exhibited marked insulin resistance. Glycemic control remained suboptimal despite treatment with a GLP-1 receptor agonist but improved dramatically following the addition of tirzepatide. This case highlights the potential therapeutic role of dual GLP-1/GIP receptor agonism in the management of metabolic disturbances associated with lipodystrophy and provides insights into its underlying mechanisms.

## Case presentation

**Figure 1:**
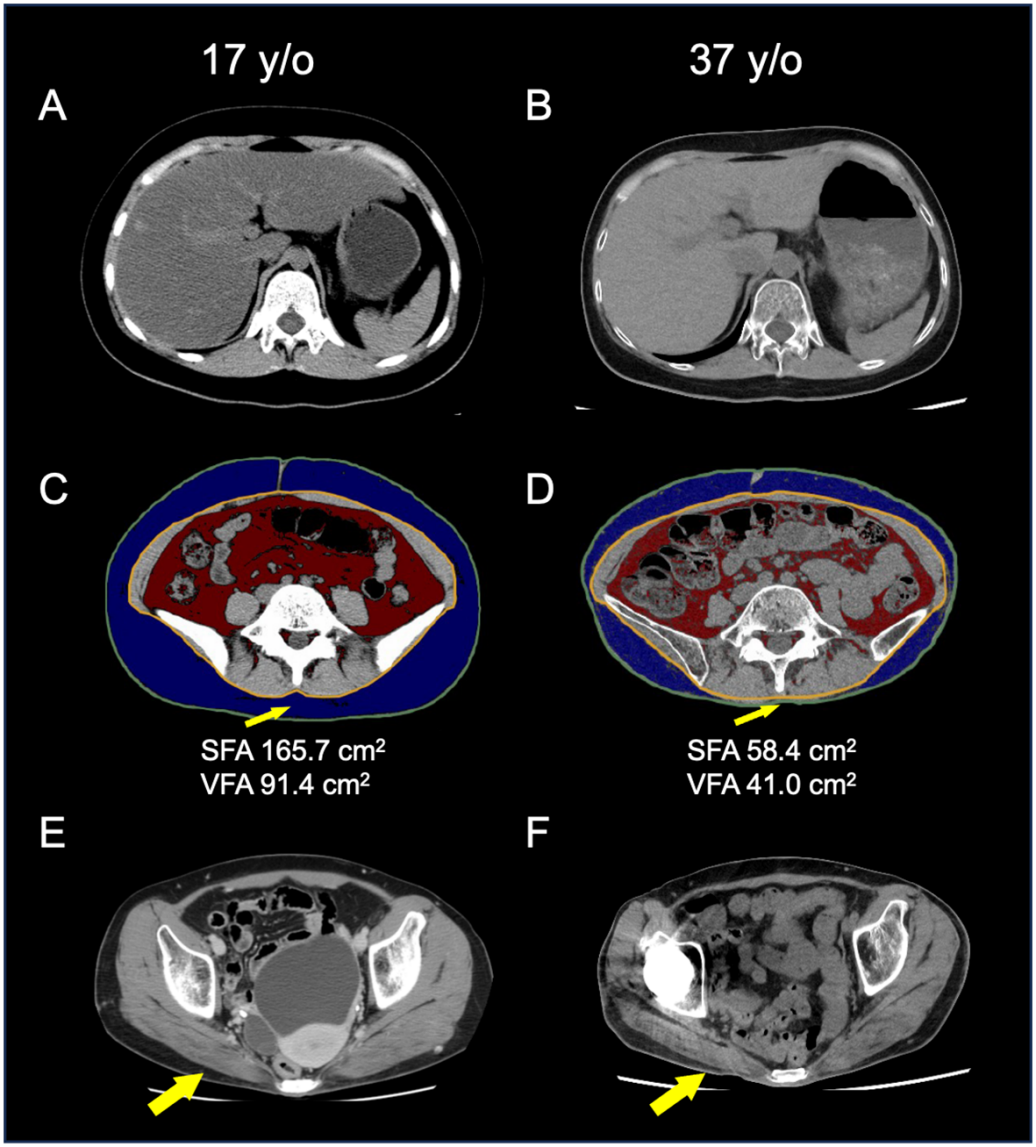
Computed tomography (CT) images demonstrating changes in fat distribution and hepatic steatosis. **(A, B)** Axial CT images at the upper abdominal level obtained at 17 **(A)** and 37 **(B)** years of age, showing improvement in hepatic steatosis. **(C, D)** Axial CT images at the umbilical level obtained at 17 **(C)** and 37 **(D)** years of age. Subcutaneous fat area (SFA) and visceral fat area (VFA) are shown. Both SFA and VFA are reduced at 37 years compared with 17 years. In panel D, an arrow indicates a reduction in subcutaneous fat in the lower back region. **(E, F)** Axial CT images at the pelvic level obtained at 17 **(E)** and 37 **(F)** years of age. Subcutaneous fat in the gluteal region is reduced at 37 years, as indicated by arrows.

**Figure 2:**
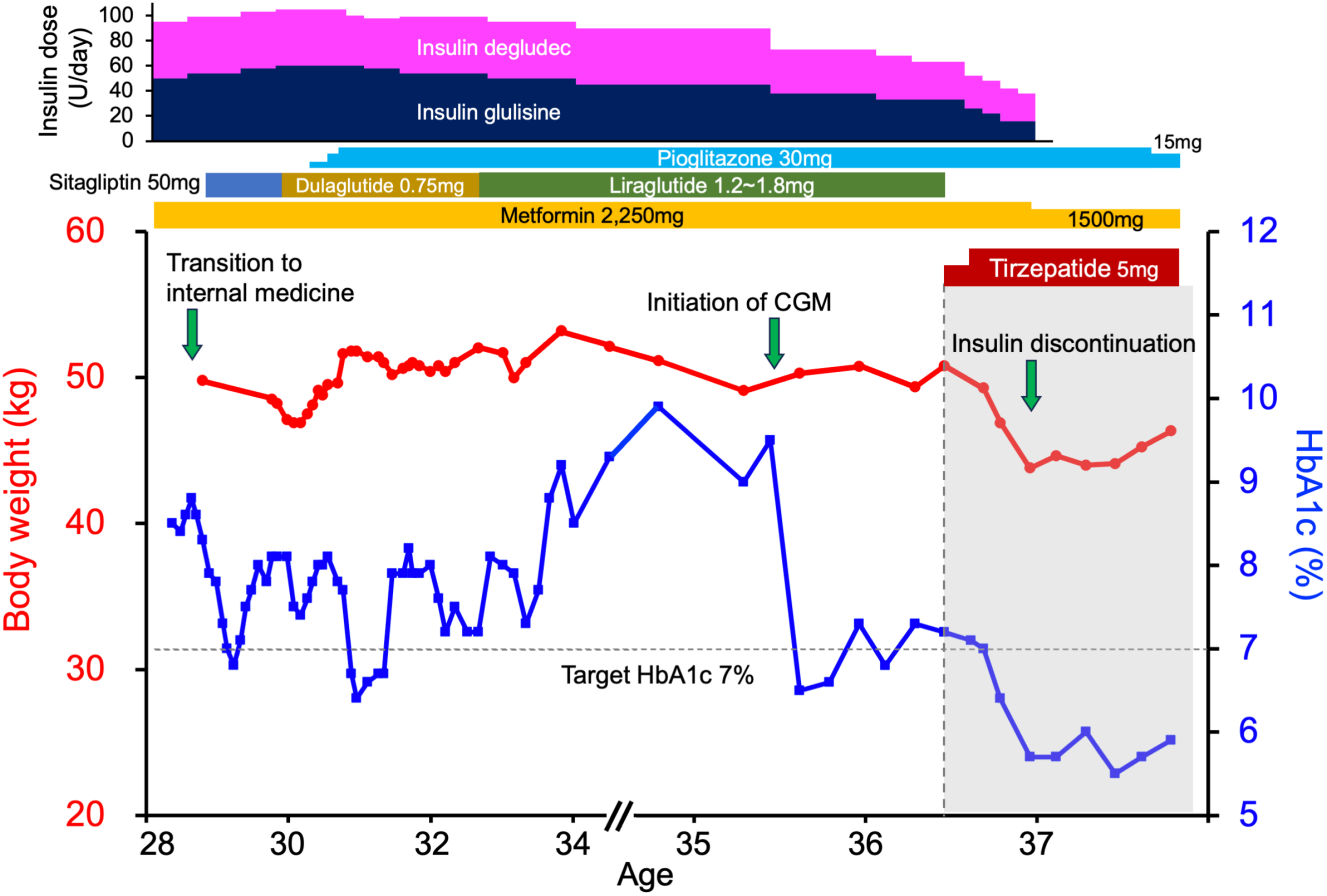
Clinical course of body weight, HbA1c, insulin dose, and pharmacological treatment. Changes in body weight (red line), HbA1c (blue line), and daily insulin dose are shown from 28 to 37 years of age. Major therapeutic interventions and antidiabetic medications are indicated above the graph. Continuous glucose monitoring (CGM) was initiated at 35 years of age. Tirzepatide therapy was initiated at 2.5 mg, increased to 5 mg after 5 weeks, and maintained thereafter. This treatment was followed by marked improvement in glycemic control and eventual discontinuation of insulin therapy.

A 37-year-old woman was diagnosed with acute lymphoblastic leukemia at 7 years of age and received chemotherapy and cranial radiotherapy. Following relapse, she underwent allogeneic HSCT at 12 years of age after a conditioning regimen including total body irradiation (TBI), high-dose cytarabine, and melphalan, and achieved complete remission. Following engraftment, she developed GVHD, which was treated with high-dose corticosteroids and weekly methotrexate. At 17 years of age, she was diagnosed with diabetes mellitus based on a postprandial plasma glucose level of 224 mg/dL, HbA1c of 6.7%, and a serum C-peptide immunoreactivity level of 11.3 ng/mL. Thyroid function tests were within normal limits. She was also found to have hyperuricemia, dyslipidemia, fatty liver, and hypogonadism. At approximately 16 years of age, she developed persistent irregular genital bleeding. Endocrine evaluation before initiation of hormone therapy revealed LH 2.7 mIU/mL, FSH 5.5 mIU/mL, and estradiol 33 pg/mL. She subsequently received long-term hormone replacement therapy, including cyclic progestin therapy during late adolescence and combined estrogen–progestin therapy in early adulthood. She currently has amenorrhea. Her body mass index (BMI) at that time was 21.1 kg/m². Abdominal computed tomography (CT) revealed fatty liver, while both subcutaneous and visceral fat in the abdominal region were preserved (**Figures 1A, C**). No apparent thinning of the subcutaneous fat in the buttocks or lower legs was observed. Treatment for diabetes was initiated with metformin and an α-glucosidase inhibitor, and insulin therapy was added at 25 years of age. At 28 years of age, her care was transitioned from the pediatric department to the internal medicine department. At that time, because she rarely consumed breakfast, she was treated with intensive insulin therapy consisting of insulin glulisine (0–15–30 units before meals) and insulin glargine (50 units at supper), totaling 95 units per day, in addition to metformin 2,250 mg/day. Her HbA1c levels remained in the high 8% range (**Figure 2**).

At 28 years old, the patient’s height, body weight, BMI, blood pressure, and pulse rate were 160 cm, 49.8 kg, 19.5 kg/m², 110/70 mmHg, and 65 beats/min respectively. Physical examination was unremarkable, and no marked regional differences in subcutaneous fat distribution were observed. She had no family history of diabetes mellitus. Laboratory findings are shown in **Table 1**. Urinary albumin excretion and serum leptin were 11 mg/g creatinine and 9.6 ng/mL, respectively.

**Table 1:** Clinical characteristics and laboratory findings during the clinical course.

Findings	28 y/o	36 y/o (Before tirzepatide)	37 y/o (After 8 months of tirzepatide therapy)	Reference range
Physical findings				
Body weight (kg)	49.8	50.8	44.6	
Body mass index (kg/m^2^)	19.5	19.8	17.4	
SBP (mmHg)	110	114	108	
DBP (mmHg)	70	72	66	
Biochemical test				
White blood cell (/μL)	8,100	6,600	4,700	3,300–8,600
Red blood cell (×10^6^/μL)	4.22	3.81	3.61	3.86–4.92
Platelet (×10^5^/μL)	3.16	2.83	2.57	1.58–3.48
AST (IU/L)	36	18	22	13–30
ALT (IU/L)	37	17	15	7–23
γ-GTP (IU/L)	165	63	37	9–32
Uric acid (mg/dL)	4.1	3.3	3.6	2.6–5.5
FPG (mg/dL)	138	139	103	73–109
HbA1c (%)	8.6	7.2	5.7	4.6–6.2
LDL cholesterol (mg/dL)	164	137	108	62–163
HDL cholesterol (mg/dL)	36.2	45.8	55.7	48–103
Triglycerides (mg/dL)	235	119	99	30–117
Endocrinology				
Adiponectin (μg/mL)	NA	NA	27.8	5–30
HMW adiponectin (μg/mL)	NA	NA	17.8	2–10
Leptin (ng/mL)	9.6	NA	3.4	4–25

All blood samples were collected following an overnight fast.

SBP Systolic blood pressure, DBP Diastolic blood pressure, AST aspartate transaminase, ALT alanine aminotransferase, γ-GTP γ-glutamyl transpeptidase, FPG fasting plasma glucose, LDL low-density lipoprotein, HDL high-density lipoprotein, HMW high-molecular-weight.

Sitagliptin, pioglitazone, dulaglutide, and liraglutide were sequentially added to the baseline regimen. Although transient improvements in HbA1c were observed, levels generally remained above 7% (**Figure 2**). The total daily insulin requirement remained approximately 90–100 units. She performed self-monitoring of blood glucose only once or twice daily until the age of 35 years, when the introduction of continuous glucose monitoring (CGM) led to an improvement in HbA1c from the 9% range to approximately 7%. However, the total daily insulin dose decreased only marginally. At 36 years of age, liraglutide was discontinued and replaced with tirzepatide 2.5 mg. The dose of tirzepatide was increased to 5 mg after 5 weeks and maintained thereafter. The insulin dose was gradually reduced from 83 units/day at baseline and was discontinued 5 months after the initiation of tirzepatide. Metformin was also reduced to 1,500 mg/day (**Figure 2**). Pioglitazone was continued but reduced to 15 mg/day during follow-up. Thereafter, HbA1c was maintained at approximately 5.7%, and time in range assessed by CGM remained around 90%. Tirzepatide was generally well tolerated. The patient experienced only mild nausea during treatment, which did not interfere with continuation of therapy, and no severe adverse events were observed.

During treatment with tirzepatide (8 months after initiation), serum leptin, adiponectin, high-molecular-weight (HMW) adiponectin, and HMW/total adiponectin ratio were 3.4 ng/mL, 27.8 μg/mL, 17.8 μg/mL, and 64.0%, respectively (**Table 1**). Body weight decreased from 50.8 kg before the initiation of tirzepatide to 44.6 kg after 8 months. On abdominal CT at the level of the umbilicus, both visceral and subcutaneous fat areas were clearly reduced compared with those at 17 years of age, and hepatic steatosis had also improved (**Figures 1A–D**). In addition, region-specific fat loss extending from the lower back to the gluteal region was observed (**Figures 1C–F**). A follow-up fasting C-peptide measurement obtained 10 months after initiation of tirzepatide showed a fasting plasma glucose level of 128 mg/dL and a serum C-peptide level of 2.8 ng/mL.

## Discussion

We describe a woman who developed severe insulin resistance accompanied by diabetes mellitus and fatty liver disease five years after HSCT. Although no apparent fat loss was initially recognized, region-specific subcutaneous fat loss was observed at 37 years of age. Glycemic control remained inadequate despite high-dose insulin therapy and a GLP-1 receptor agonist, whereas treatment with the dual GIP/GLP-1 receptor agonist tirzepatide led to marked metabolic improvement and discontinuation of insulin therapy. This striking therapeutic response raises important questions regarding the underlying mechanisms of her metabolic dysfunction.

The diagnosis of lipodystrophy syndromes is primarily clinical, as universally accepted diagnostic criteria have not been established. International guidelines recommend a composite assessment based on characteristic fat distribution, metabolic abnormalities such as severe insulin resistance and hepatic steatosis, supportive imaging findings, and exclusion of alternative causes (1). Notably, no specific diagnostic criteria have been proposed for lipodystrophy occurring after HSCT, and post-transplant cases are generally diagnosed based on clinical judgment (3). In the present case, the patient underwent HSCT at 12 years of age and was first diagnosed with diabetes mellitus and fatty liver at 17 years. At that time, CT demonstrated preserved abdominal subcutaneous and visceral adipose tissue, and no apparent thinning of subcutaneous fat in the gluteal region or lower extremities was observed. No apparent partial lipoatrophy was recognized at the time of transfer to our care at 28 years of age. However, CT at 37 years of age revealed evident subcutaneous fat loss extending from the lower back to the gluteal region. The delayed appearance of clinically evident lipoatrophy is also noteworthy. Previous reports have suggested that post-HSCT lipodystrophy may become apparent many years after transplantation, potentially reflecting progressive adipose tissue remodeling and chronic immune-mediated injury (17–19). The evolution of fat loss observed in our patient is consistent with this proposed mechanism. Given the marked insulin resistance despite a non-obese phenotype, together with the site-specific reduction in subcutaneous fat, this case was considered to represent acquired partial lipodystrophy. This patient developed GVHD following HSCT and received intensive immunosuppressive therapy, including high-dose corticosteroids and methotrexate. GVHD is characterized by immune-mediated tissue injury and chronic inflammation, which may affect adipose tissue distribution and function. Previous reports have suggested that acquired partial lipodystrophy can occur as a manifestation of GVHD (18, 19). Therefore, GVHD-associated immune dysregulation may have contributed to adipose tissue dysfunction in this case. Taken together, these findings suggest a lipodystrophy-like metabolic phenotype developing after HSCT, although the lack of standardized diagnostic criteria for post-transplant lipodystrophy limits definitive classification. Importantly, this patient received TBI as part of the HSCT conditioning regimen. Recent studies have suggested that TBI-based conditioning may represent an important risk factor for the later development of acquired partial lipodystrophy after childhood HSCT (6, 10, 18). TBI-induced adipocyte injury, together with chronic GVHD-associated inflammation and long-term immunosuppressive therapy, may have contributed to the delayed development of adipose tissue dysfunction and regional fat loss in this patient.

Recombinant leptin replacement therapy has been reported to improve metabolic parameters in patients with lipodystrophy, particularly in those with generalized forms or with demonstrable leptin deficiency. The therapeutic effects of leptin are thought to result from central and peripheral actions that enhance insulin sensitivity, decrease hepatic steatosis, and reduce ectopic lipid accumulation by modulating appetite, energy expenditure, and lipid metabolism (20). In contrast to many previously reported cases, the patient in the present report exhibited serum leptin levels within the normal range on multiple measurements. These consistently normal values, although not shown in detail, suggest that leptin deficiency was unlikely to be the primary driver of her metabolic phenotype.

GLP-1 receptor agonists have also been described as beneficial in some cases of lipodystrophy-associated diabetes. The proposed mechanisms include enhancement of glucose-dependent insulin secretion, suppression of inappropriate glucagon release, delay of gastric emptying, and potential improvement in peripheral insulin sensitivity (21). Several clinical reports have documented modest improvements in glycemic control and reductions in insulin requirements with use of GLP-1 receptor agonists in patients with partial lipodystrophy (11, 12). However, in the present case, sequential treatment with various GLP-1 receptor agonists produced only minimal improvement in glycemic control, despite optimized dosing and concomitant insulin therapy. This limited efficacy may reflect the severity of insulin resistance associated with adipose tissue dysfunction and ectopic lipid deposition such as hepatic steatosis, or potential differences in incretin signaling in this patient. These observations underscore the heterogeneous therapeutic responses seen in lipodystrophy and highlight the need for individualized treatment strategies.

In the present case, sequential treatment with several GLP-1 receptor agonists resulted in only minimal metabolic improvement, whereas initiation of tirzepatide led to a dramatic reduction in glycemia and complete discontinuation of insulin therapy. This marked difference in therapeutic response suggests that dual incretin receptor activation, particularly sustained stimulation of the GIP receptor, may have played a critical role. Although GIP has historically been considered less effective in individuals with type 2 diabetes due to impaired incretin responsiveness, emerging experimental data indicate that pharmacologic GIP receptor agonism—especially when combined with GLP-1 receptor activation—exerts pleiotropic metabolic effects (22–25). Unlike GLP-1 receptors, which are expressed predominantly in pancreatic islets and the central nervous system, GIP receptors are highly expressed in adipocytes. Therefore, pharmacological GIP receptor agonism may exert direct effects on adipose tissue biology beyond appetite suppression and weight reduction. Preclinical studies have demonstrated that GIP receptor signaling influences adipocyte lipid handling, enhances insulin sensitivity in adipose tissue, and modulates systemic lipid partitioning (13). In lipodystrophy, where adipose tissue dysfunction leads to ectopic lipid accumulation in the liver and skeletal muscle, restoration of adipocyte metabolic competence could theoretically reduce lipotoxicity and improve whole-body insulin resistance. The preserved fasting C-peptide level (2.8 ng/mL) observed after discontinuation of insulin therapy is consistent with the possibility that improved insulin sensitivity contributed importantly to the metabolic benefits of tirzepatide.

One plausible explanation for the observed improvement is the substantial weight reduction following tirzepatide initiation. Weight loss is known to decrease hepatic fat content, reduce inflammatory signaling, and improve insulin sensitivity at both hepatic and peripheral levels (26). In patients with fatty liver disease, even modest reductions in body weight are associated with significant improvements in hepatic steatosis and glycemic control (27). Therefore, part of the therapeutic benefit in this case may be attributable to weight loss–mediated reductions in hepatic lipid burden and consequent improvement in insulin resistance.

However, accumulating evidence suggests that tirzepatide exerts metabolic effects beyond those explained by weight reduction alone. Experimental models have shown that GIP receptor activation may enhance adipose tissue blood flow, improve triglyceride clearance, and promote more efficient lipid storage within subcutaneous depots, thereby limiting ectopic fat deposition (13, 28). Dual GIP/GLP-1 receptor agonism has also been reported to improve insulin sensitivity independently of body weight changes, potentially through direct actions on adipocytes, skeletal muscle, and hepatic metabolism (28). In the context of post-transplant acquired partial lipodystrophy, where adipose tissue quantity may appear relatively preserved but functional impairment is suspected, sustained GIP receptor stimulation could have enhanced residual adipocyte function and improved lipid buffering capacity. This mechanism may explain the marked improvement in insulin resistance and glycemic control observed in our patient, despite only partial changes in overall adiposity.

Taken together, the present case raises the possibility that GIP receptor agonism contributes uniquely to metabolic restoration in lipodystrophy-associated diabetes, through both weight-dependent and weight-independent mechanisms. Further mechanistic and clinical studies are warranted to clarify the role of dual incretin receptor activation in disorders characterized by adipose tissue dysfunction.

Adiponectin is an adipocyte-derived hormone with insulin-sensitizing, anti-inflammatory, and anti-steatotic properties, and its HMW isoform is believed to be the most biologically active (29). In healthy adults, the HMW/total adiponectin ratio typically ranges from 30–60%, with lower ratios being associated with increased insulin resistance and metabolic dysfunction (30). In metabolic disorders such as obesity, type 2 diabetes, and nonalcoholic fatty liver disease, both circulating total adiponectin and the HMW fraction are often reduced, and a decreased HMW/total adiponectin ratio correlates with greater severity of insulin resistance, systemic inflammation, and ectopic fat deposition (29). Interventions that improve adipose tissue function, including lifestyle-induced weight loss and certain pharmacologic treatments, have been shown to increase total adiponectin levels and the HMW/total adiponectin ratio. For example, dietary weight loss in overweight and obese adults increases the HMW adiponectin fraction and HMW/total ratio, concomitant with improvements in insulin sensitivity (31). Similarly, pharmacologic therapies such as thiazolidinediones have been reported to raise adiponectin multimer levels and enhance the HMW/total ratio in patients with type 2 diabetes, supporting the concept that adipose tissue function can be modulated independently of absolute weight change (28, 32).

In the current case, we observed an elevated HMW/total adiponectin ratio (64.0%) following tirzepatide therapy. The post-treatment HMW adiponectin level (17.8 μg/mL) and HMW/total adiponectin ratio (64.0%) were at the upper end of reported ranges despite severe insulin resistance and a history of acquired partial lipodystrophy (29, 30). This observation raises the possibility that tirzepatide may exert beneficial effects on adipose tissue secretory function beyond its weight-lowering actions. Although tirzepatide treatment has been reported to increase total adiponectin levels in patients with type 2 diabetes (32–35), data regarding HMW adiponectin or the HMW/total ratio are lacking, including in patients with type 2 diabetes as well as in those with lipodystrophy or related severe metabolic diseases. Given that alterations in adiponectin multimerization have been linked to improvements in systemic insulin sensitivity independent of weight loss, the elevated HMW/total adiponectin ratio observed in our patient may reflect a qualitative improvement in adipose tissue function mediated by dual incretin receptor activation. However, this interpretation remains speculative because baseline adiponectin measurements were unavailable and direct evidence for tirzepatide-induced increases in the HMW/total adiponectin ratio in humans is currently lacking. Prospective studies incorporating baseline and longitudinal adiponectin multimer measurements are needed to clarify these potential mechanisms.

The patient was highly satisfied with the clinical outcome, particularly because insulin therapy could be discontinued. However, given the relatively low BMI achieved after treatment, dose reduction of tirzepatide is currently being considered to prevent excessive weight loss while maintaining metabolic control.

## Limitations

Several limitations of the present report should be acknowledged. First, although evident site-specific fat loss was observed at 37 years of age, the timing of its onset remains unclear. Second, the marked metabolic improvement observed after initiation of tirzepatide may have been partially attributable to weight reduction. However, the magnitude of improvement in insulin resistance and the rapid discontinuation of high-dose insulin therapy suggest that mechanisms beyond weight loss alone may have contributed. Finally, adiponectin and HMW adiponectin levels were not measured prior to tirzepatide initiation, and follow-up C-peptide measurements were available only after tirzepatide initiation. These limitations highlight the need for further studies to clarify the mechanisms underlying metabolic improvement with dual incretin receptor agonism in lipodystrophy-like conditions.

## Conclusion

This case highlights acquired partial lipodystrophy developing after HSCT, characterized by region-specific subcutaneous fat loss in the lower back and gluteal regions, together with severe insulin resistance and fatty liver disease. HSCT and its associated immune and inflammatory sequelae, including graft-versus-host disease, may adversely influence adipose tissue biology and contribute to the development of acquired lipodystrophy.

Despite minimal response to GLP-1 receptor agonists, dual GIP/GLP-1 receptor agonism with tirzepatide resulted in marked metabolic improvement and discontinuation of high-dose insulin therapy. While part of this effect may be attributable to weight reduction, weight-independent mechanisms—potentially involving restoration of adipose tissue function—are likely to have contributed.

The elevated HMW/total adiponectin ratio observed after tirzepatide treatment raises the possibility of improved adipocyte secretory capacity, although baseline adiponectin levels were not available and causality cannot be established. This case suggests that dual incretin receptor agonism may represent a promising therapeutic strategy for severe insulin resistance associated with acquired lipodystrophy.

## Data Availability

The raw data supporting the conclusions of this article will be made available by the authors, without undue reservation.
